# 

**Published:** 1886-11

**Authors:** 


### CONTENTS—NOVEMBER, 1886.

ORIGINAL COMMUNICATIONS—Absorption of Cataract—One Year after First Discission, and ten months after second By T. J. Tyner, M. D., San Antonio...... ........ 181
Strychnia Hypodermically in Malarial Hæmaturia. By S. Leard Keown, M. D., Dallas, Texas............ 182
A Case of Inertia Uteri. C, L. Gwyn, M. D., Galveston 188
A Case of Compound Comminuted Fracture of the Frontal Bone, with Depression, Recovery. Eugene Clark, M. D., Lockhart, Texas.................. 189
CORRESPONDENCE—Tait's Operation in Texas. Letter from Dr. C. H. Wilkinson, Galveston ............ ... 191
Tait's Operation in Texas, Letter from Dr. Baldwin of Ohio,........................................ 192
Letter from Dr. E. J. B. Ft. Worth .................. 193
Letter from Dr. A. Wadgymar, Corrizo Springs........ 193
"Misguided Members of the Profession" and the Presidential Addresses again. Action of the East Line (Dr. Becton's Home) Association........................ 193
Rare effects of Quinine on the Skin. Letter from J. E. Roach, M. D., Sipe Springs...................... 195
Dislocation of Occipital Bone: Letter from Dr. W. H. Lancaster, Coleman, Texas.................... 197
A Unique Case of Minor Surgery. Letter from Dr. W. A. Barton, Newport, Texas...................... 198
A New Method in Incarcerated Scrotal Hernia. Note from Dr. W. A. Lockett, Brenham............... 198
SOCIETY NOTES—The Texas State Medical Association.... 199
Ninth International Medical Congress ............. 200
Notice from Secretary T. S. M. A. to Members ...... 202
EDITORIAL—The Surgeon's Relation to Homicides before the Courts........................................ 203
In the Interest of Peace .................... 206
Requisites of a Gynæcologist. The Michigan Ideal.. 208
Biography of Contemporary Physicians of Texas...... 209
MEDICAL NEWS AND MISCELLANY...................... 210
BIBLIOGRAPHY.......................................... 215
ADVERTISERS NOTICES.......................... 217

